# Potential Application of Human β-Defensin 4 in Dental Pulp Repair

**DOI:** 10.3389/fphys.2020.01077

**Published:** 2020-08-21

**Authors:** Yue Zhai, Xiaojing Yuan, Yuming Zhao, Lihong Ge, Yuanyuan Wang

**Affiliations:** Department of Pediatric Dentistry, Peking University School and Hospital of Stomatology, Beijing, China

**Keywords:** human β-defensin 4, dental pulp stem cells, anti-inflammatory, stem cell differentiation, vital pulp therapy

## Abstract

When pulp tissue is damaged by caries or trauma, vital pulp therapy (VPT) can help preserve the pulp tissue for long-term retention of teeth. However, the choice of pulp capping agent used in VPT is important for the successful preservation of the pulp tissue. Here we investigated the expression and biological function of human β-defensin 4 (HBD4) in dental pulp stem cells (DPSC) and explored its potential as a pulp capping agent. We examined the expression of HBD4 in DPSC *in vitro* using qPCR and immunofluorescence staining. We also looked at the effect of HBD4 on inflammatory factors in lipopolysaccharide (LPS)-stimulated DPSC, and its effects on mineralizing cell phenotype differentiation, via qPCR and western blot. Finally, we examined the ability of HBD4 to promote the restoration of the pulp-dentin complex *in vivo*, using male Wistar rats with reversible pulpitis. We found HBD4 was highly expressed in DPSC stimulated by TNF-α and IL-1α. HBD4 down-regulated the expression of inflammatory mediators (i.e., IL-1α, IL-1β, IL-6, TNF-α) in LPS-stimulated DPSC, and suppressed MAPK activity and the NF-κB pathway. HBD4 also enhanced the differentiation of DPSC into osteoblasts or odontoblasts, potentially by modulating the Notch pathway. Furthermore, HBD4 controlled the degree of pulp inflammation in a rat model of reversible pulpitis and induced the formation of restorative dentin. Together our findings indicate HBD4 may be a useful pulp capping agent for use in VPT.

## Introduction

About 2.43 billion people (36% of the world’s population) have permanent tooth caries (Vos et al., 2012). Shallow caries can be treated by filling, but for teeth with deep caries or severe trauma, the pulp tissue is easily infected, causing inflammation and pain. If untreated, the infection may spread to the entire pulp or surrounding tissue, and may eventually require root canal treatment or tooth extraction.

Although root canal therapy is an effective way to treat pulpitis and prevent periapical periodontitis (Kojima et al., 2004), clinical studies have shown the long-term survival rate of teeth after root canal therapy is lower, especially in molars. Root canal therapy may reduce the fracture resistance of root, which is an important cause of vertical root fracture. The possible reason is that teeth lose their proprioceptive, damping, and sensitive functions after root canal treatment, which are required for the detection of harmful stimuli and subsequent defense (Seo et al., 2012; Russell et al., 2014; Mikrogeorgis et al., 2018). In addition, root canal therapy is expensive, and patients often need multiple visits. Alternatively, vital pulp therapy (VPT) can avoid damaging the pulp-dentin complex and helps preserve the remaining pulp tissue (Saghiri et al., 2016). VPT is particularly important for young permanent teeth with incomplete apical foramen, as the remaining vital pulp can promote root development. Therefore, VPT should be performed as often as possible.

Mineral trioxide aggregate (MTA) is currently considered to be a choice for VPT, due to its capacity of biocompatibility, sealing properties, and ability of inducing mineralized tissue formation. However, in treatment of chronic diffuse pulpitis, MTA falls short of strong antibacterial and anti-inflammatory properties, leaving the possibility of infection recurrence and subsequent death of the whole pulp tissue (Kim et al., 2018). Another notable limitation of MTA is the requirement of exposing the pulp to high pH to catalyze the formation of the pulp-dentin complex, and/or the resulting dentin bridge is permeable and susceptible to bacterial infection (Li et al., 2015). Such unwanted effects may be alleviated if we were able to better control the homing and differentiation of stem cells that are used to repair the pulp-dentin complex (Schmalz and Smith, 2014). Indeed, based on our improved understanding of the natural restoration process of the pulp-dentin complex, some researchers have tried to treat pulp diseases by using biological methods to form regenerative tissue (Iohara et al., 2004). In addition, certain bioactive proteins may help to reduce the initial inflammation, attract stem cells in the pulp to the damaged site, stimulate stem cell proliferation, induce stem cells to differentiate into odontoblasts, and promote the deposition of restorative dentin; however, the roles of such bioactive proteins have only recently been characterized in the formation of hard tissues, and their anti-inflammatory and antibacterial functions are unclear (Wang et al., 2018).

Human β defensin 4 (HBD4) is a small positively-charged polypeptide (net charge of + 6), containing a three-stranded reverse-parallel β-sheet structure at its core, stabilized by three disulfide bonds (García et al., 2001). HBD4 is expressed in gingiva (Li et al., 2016), cartilage (Musumeci et al., 2012), skin, lung (Yanagi et al., 2005), testis, uterus, kidney, and other tissues (Otte et al., 2009). In addition, HBD4 has been shown to have antibacterial activity (Sharma and Nagaraj, 2012), including against some drug-resistant strains, such as Acinetobacter baumannii (Supp et al., 2009). We previously showed HBD4 was highly expressed in stem cells derived from human exfoliated deciduous teeth (SHED) stimulated by proinflammatory cytokines, and possessed anti-inflammatory and antibacterial activity. Moreover, we found HBD4 promoted osteogenic/odontogenic differentiation of SHED. Therefore, HBD4 may represent a suitable agent for VPT in primary teeth (Zhai et al., 2019).

In this study, we examine the expression and function of HBD4 in dental pulp stem cells (DPSC) to further explore whether HBD4 could be used for vital pulp preservation of permanent teeth. In particular, we aimed to: (1) evaluate the expression of HBD4 in DPSC; (2) determine whether HBD4 can inhibit lipopolysaccharide (LPS)-mediated inflammation in DPSC; (3) explore whether HBD4 can induce DPSC to differentiate into osteoblasts/odontoblasts; and (4) test whether HBD4 can promote the restoration of the pulp-dentin complex in a rat model of reversible pulpitis.

## Materials and Methods

### Cell Culture and Characterization

DPSC were donated by the Oral Stem Cell Bank of Beijing, Tason Biotech Co., Ltd. (Beijing, China), derived from three different individuals. Cells were then cultured in α-minimum essential medium (α-MEM, Hyclone, Logan, UT, United States) supplemented with 10% fetal bovine serum (FBS, Gibco, Mulgrave, VIC, Australia), 100 U/mL penicillin, and 100 μg/mL streptomycin (Solarbio, Beijing, China) in a humidified atmosphere of 5% CO_2_ at 37°C. The cells used in experiments were between passage 3 and 5.

The surface markers of DPSC were identified by flow cytometry. Cells were detached with trypsin/ethylenediaminetetraacetic acid (Gibco) to produce single-cell suspension and resuspended in phosphate buffered saline (PBS) containing 2% FBS. Cells at a concentration of 1 × 10^6^ cells/mL were then incubated with the following monoclonal antibodies: CD34-PE, CD45-PE, CD73-PE, CD90-FITC, CD105-FITC, and CD146-PE (BD Pharmingen, San Diego, CA, United States). The stained cells were analyzed using the flow cytometry system (FC500, Beckman Coulter, Brea, CA, United States) to detect fluorescence intensity and positive rate.

### Differentiation of DPSC Into Multiple Lineages

To test the stemness of DPSC, we examined their osteogenic, adipogenic, and chondrogenic differentiation ability. To induce osteogenic differentiation, cells were incubated in osteogenic induction medium (α-MEM containing 10 mM β-glycerophosphate, 50 mg/L ascorbic acid, 10 nM dexamethasone, and 10% FBS) for 21 days. The mineralization was detected by staining with 2% w/v Alizarin Red S. To induce adipogenic differentiation, cells were incubated in adipogenic induction medium (α-MEM containing 0.5 mM 3-isobutyl-1-methylxanthine, 100 μM indomethacin, 1 mg/mL insulin, 1 mM dexamethasone, and 10% FBS) for 14 days. Lipid droplets were stained with 0.5% w/v Oil Red O reagent (Jo et al., 2007). To induce chondrogenic differentiation, 4 × 10^5^ cells were transferred to a centrifuge tube and centrifuged at 1000 rpm for 4 min. The supernatant was aspirated and 0.5 mL of cartilage induction medium (Cyagen, Suzhou, China) was added. Cells were cultured at 37°C and 5% CO_2_ for 28 days (Hou et al., 2015). The “cartilage pellet” was fixed and paraffin-embedded, and alicin blue staining was performed.

### Quantitative Polymerase Chain Reaction

RNA was isolated using TRIzol (Invitrogen, Carlsbad, CA, United States) following the manufacturer’s instructions. Reverse transcription of RNA was performed using the transcriptor first-strand complementary DNA synthesis kit (Takara Biotechnology, Dalian, Liaoning, China). The primers used in the quantitative polymerase chain reaction (qPCR) analysis are outlined in Table 1. The qPCR with total RNA was performed with a SYBR Green System (7300 Real Time System, Applied Biosystems, Carlsbad, CA, United States) according to the manufacturer’s protocol. Target gene expressions were normalized with β-actin. Relative gene expression values were calculated by ΔΔCT-based fold-change calculations. The qPCR products of HBD1-4 were resolved on a 1.5% agarose gel and stained with SYBR Green I (Solarbio).

**TABLE 1:** Primers used for qPCR.

Target gene	Sequence	Product size (bp)	GenBank number
β-actin	Forward: CCTGGCACCCAGCACAAT	144	NM_001101.5
	Reverse: GGGCCGGACTCGTCATACT		
HBD1	Forward: CATGAGAACTTCCTACCTTCTGC	208	NM_005218.4
	Reverse: TCACTTGCAGCACTTGGCCTT		
HBD2	Forward: ATCAGCCATGAGGGTCTTGT	172	AF040153
	Reverse: GAGACCACAGGTGCCAATTT		
HBD3	Forward: AGCCTAGCAGCTATGAGGATC	206	NM_018661.4
	Reverse: CTTCGGCAGCATTTTCGGCCA		
HBD4	Forward: TGCCTTAAGAGTGGAGCCATA	109	NM_004942
	Reverse: CTCCTCATGGCTTTTTGCAG		
TNF-α	Forward: GGCTCCAGGCGGTGCTTGTTC	190	NM_000594.4
	Reverse: CAGGCTTGTCACTCGGGGTTCG		
IL-6	Forward: GGTGTTGCCTGCTGCCTTCC	193	NM_000600.5
	Reverse: TGCCTCTTTGCTGCTTTCACAC		
IL-1α	Forward: TGAAGGCAAAGCACGAAATGTTAT	198	NM_000575.4
	Reverse: TGGACCAAAATGCCCTGTAT		
IL-1β	Forward: GGCAGGCCGCGTCAGTTG	198	NM_000576.2
	Reverse: CCCGGAGCGTGCAGTTCAGT		
TLR2	Forward: GCGTGGCCAGCAGGTTCAGG	167	XM_011532216.2
	Reverse: GGAGCCAGGCCCACATCATTTTC		
TLR4	Forward: TGCAATGGATCAAGGACCAG	147	NM_138554.5
	Reverse: TGAGGACCGACACACCAATG		
Runx-2	Forward: CTGAGGTAACTTGCTAACG	101	NM_001024630.4
	Reverse: ATCAATACACTAAGAAATGTTTCAAGG		
OCN	Forward: AGCAAAGGTGCAGCCTTTGT	261	NM_199173.5
	Reverse: GCGCCTGGGTCTCTTCACT		
DMP-1	Forward: TGGGGATTATCCTGTGCTCT	129	XM_011531706.2
	Reverse: GCTGTCACTGGGGTCTTCAT		
DSPP	Forward: TCCTAGCAAGATCAAATGTGTCAGT	152	NM_014208.3
	Reverse: CATGCACCAGGACACCACTT		

### Immunofluorescence Staining

DPSC were seeded into 12-well plates. After the corresponding treatment, DPSC were fixed in 4% paraformaldehyde for 10 min, permeabilized with 0.1% Triton X-100 for 5 min, and blocked with 5% BSA for 2 h. Cells were then incubated with the corresponding primary antibody [i.e., anti-HBD4 (1:400; Abcam, Cambridge, United Kingdom) or anti-p65 (1:500; Cell Signaling Technology, Beverly, MA, United States)] overnight at 4°C, before incubating with the anti-rabbit secondary antibody (1:500; Abcam, Cambridge, United Kingdom) for 1 h at room temperature. Nuclei were counterstained with 4′,6-diamidino-2-phenylindole (DAPI, Solarbio) and the coverslips were mounted on a glass slide. Images were captured using a fluorescence microscope (Nikon, Japan).

### Cell Viability and Transwell Migration Assays

Cell viability assays were performed using the Cell Counting Kit-8 (Beyotime, Shanghai, China). DPSC at a concentration of 5 × 10^4^ cells/mL were plated in 96-well plates. After overnight incubation, the cells were treated with HBD4 (Beyotime) for the indicated times. A total of 10 μL CCK-8 solution was then added to each well. After incubation for 1 h at 37°C, the optical density (OD) value of each well was measured at a wavelength of 450 nm. The experiments were performed in triplicate.

For the transwell migration assays, DPSC were cultured in serum-free medium overnight. A total of 100 μL 5 × 10^4^ cells were seeded in the upper chamber of a transwell culture plate (8 μm pore size, 24-well; Corning, CA, United States). Four groups were set in the bottom chamber: control, 10% FBS, 20 μg/mL HBD4, and 40 μg/mL HBD4. Cells were incubated for 24 h, fixed with methanol, and stained with Giemsa Stain solution (Solarbio). Cells on the underside of the membrane were stained and counted under a microscope in three random fields. The experiments were performed in triplicate (Alqahtani et al., 2018; Cheng et al., 2019).

### Western Blot

Cells were harvested and lysed in RIPA lysis buffer with phenylmethylsulphfonyl fluoride (Beyotime). Protein concentrations were detected using the Pierce BCA Protein Assay Kit (Thermo Fisher Scientific, Waltham, MA, United States). The protein samples were separated on sodium dodecyl sulfate polyacrylamide gel (SDS-PAGE) and transferred to polyvinylidene difluoride (PVDF) membranes. The membranes were incubated with the corresponding primary antibodies: Runx-2 (1:1000), p42/44 mitogen-activated protein kinase (MAPK; 1:1000), phospho-p42/44 MAPK (1:2000), p38 MAPK (1:1000), phospho-p38 MAPK (1:1000), p65 nuclear factor kappa-B (NF-κB; 1:1000), phospho-p65 NF-κB (1:1000), hairy and enhancer of split-1 (HES1; 1:1000), β-actin (1:10,000; all from Cell Signaling Technology), dentin sialophosphoprotein (DSPP; 1:500; Santa Cruz Biotechnology, Santa Cruz, California, United States), and dentin matrix acidic phosphoprotein 1 (DMP-1; 1:500 Santa Cruz Biotechnology) at 4°C overnight. Subsequently, the membranes were incubated with corresponding secondary antibodies for 1 h. The blotted bands were detected using electrochemiluminescence (ECL) Western Blotting Substrate (Solarbio).

### Alkaline Phosphatase and Alizarin Red Staining

The cells were cultured in four groups: osteogenic induction medium, osteogenic induction medium + 10 μg/mL HBD4, osteogenic induction medium + 1 μg/mL LPS, osteogenic induction medium + 1 μg/mL LPS + 10 μg/mL HBD4. The concentration of LPS used in this study was based on previous data in our laboratory (Zhai et al., 2019). Alkaline phosphatase (ALP) activities of DPSC in the different groups were measured after 7 days incubation with the Alkaline Phosphatase Assay Kit (Beyotime) following the manufacturer’s protocol. The quantitative ALP activity was determined by measuring the OD values at 405 nm after incubation with p-nitrophenyl phosphate (Beyotime). After culturing for 21 days, the cells were fixed and stained with 2% Alizarin Red S (ARS; Sigma-Aldrich) solution and photographs were taken. The stained cells were then destained with 10% acetylpyridinium chloride (Sigma-Aldrich) and OD values at 590 nm were measured (Fang et al., 2019).

### Animal Model and Groups

The research was approved by the Ethics Committee of the Peking University Health Science Center (LA2019207). Fifteen 8-week-old male Wistar rats (Weitonglihua, China) were selected. After weighing, the rats were anesthetized by intraperitoneal injection of 30 g/L chloral hydrate (3 mL/kg). The rats were fixed on the operating plate in the supine position. A dental high-speed turbine handpiece was used to drill holes in the center of the bilateral maxillary first molar surface of the rats to expose the pulp chamber. Cotton filled with 1 μg/mL LPS was placed over the exposed pulp chamber for 15 min to establish the experimental pulpitis model of rats. After washing the exposed pulp chamber with saline, the rats were divided into three groups according to the different pulp capping materials used on the left maxillary first molar: the gelatin sponge (GS) group (about 1 mm × 1 mm × 1 mm), the GS + HBD4 group (1 μg/μL HBD4), and the MTA group with 5 rats in each group. The right maxillary first molar was used as a blank control. Finally, all cavities were filled with Fuji IX glass ionomer. At week 8 after the operation, the experimental teeth were obtained, fixed, decalcified, embedded, and sectioned. All experiments were completed by the same experimenter.

### Histological and Immunohistochemical Analysis

For hematoxylin-eosin (HE) staining, all paraffin sections were deparaffinized, rehydrated, stained with hematoxylin staining solution (Solarbio) for 5 min and then with eosin for 1 min, washed, dehydrated, and sealed. The damage of the pulp structure is considered as the greatly direct reflection of the pulp inflammation. The continuity of the pulp tissue, the morphology of the pulp cells and the formation of reparative dentin were observed under the light microscope to comprehensively evaluate the effect of pulp inflammation control. The histologic features were evaluated according to the criteria presented in Table 3 (Shi et al., 2016; Long et al., 2017).

For Masson staining, the sections were deparaffinized, stained with Weigert’s hematoxylin (Solarbio) for 10 min, differentiated with the acid alcohol differentiation solution for 10 s, and then washed. Finally, the sections were stained with Masson blue solution for 5 min, fuchsin staining solution for 10 min, aniline blue staining solution for 2 min, dehydrated, and sealed.

For the immunohistochemical analysis, the sections were deparaffinized, rehydrated, and subjected to Tris–EDTA (pH 9.0) antigen retrieval solution (Zhongshan Golden Bridge Biotechnology, Beijing, China) in a microwave for 30 min. Next, 3% hydrogen peroxide (Zhongshan Golden Bridge Biotechnology, Beijing, China) was used to block the endogenous peroxide. Then, the sections were incubated with either the DSPP (1:200; Santa Cruz Biotechnology) primary antibody or the vascular endothelial growth factor (VEGF, 1:500; Cell Signaling Technology) primary antibody at 4°C overnight. After a thorough rinse with PBS, sections were incubated with a secondary antibody using a horseradish peroxidase polymer system (Zhongshan Golden Bridge Biotechnology) at room temperature for 30 min. The detection step was performed with diaminobenzidine (Zhongshan Golden Bridge Biotechnology). Finally, sections were counterstained with hematoxylin to distinguish cell nuclei (Rao et al., 2019).

### Statistical Analysis

All statistical analyses were performed using SPSS 17.0 (SPSS Inc., Chicago, IL, United States) and data are presented as the mean ± standard deviation (SD). Comparisons between two groups were analyzed using the *t*-test. One-way analysis of variance was applied for multiple comparisons. The histologic evaluation results were analyzed statistically using Mann-Whitney *U*-tests. A value of *P* ≤ 0.05 was considered significant.

## Results

### DPSC Are Multipotent

**FIGURE 1:**
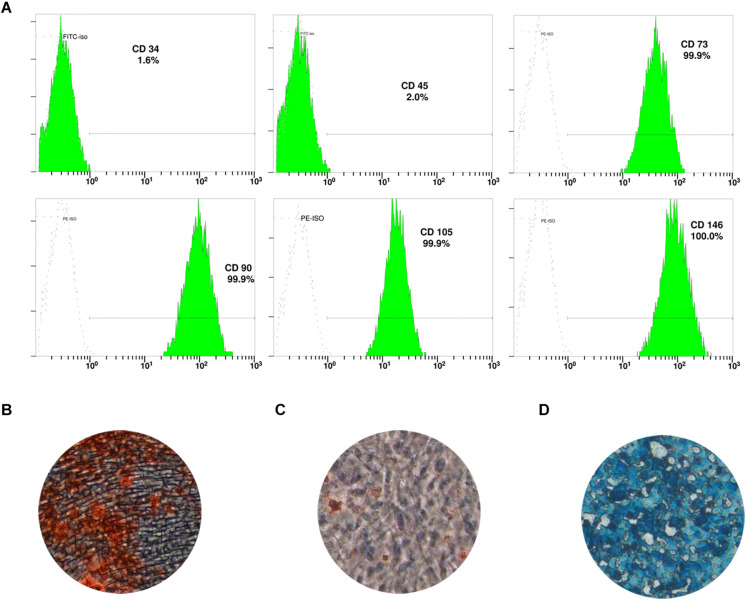
Characterization of dental pulp stem cells (DPSC). **(A)** Expression of DPSC surface markers. DPSC could be induced to form mineralized nodules **(B)**, lipid droplets **(C)**, and chondrocytes **(D)**.

DPSC showed positive expression of the mesenchymal stem cell (MSC) markers CD73 (99.9%), CD90 (99.9%), CD105 (99.9%), and CD146 (100%), but expressed low levels of the hematopoietic cell markers CD34 (1.6%) and CD45 (2.0%; Figure 1A). In addition, DPSC had the ability to differentiate into multiple lineages; in particular, they could be induced to form condensed nodules (i.e., osteogenic differentiation; Figure 1B), lipid droplets (i.e., adipogenic differentiation; Figure 1C), or chondrocytes (chondrogenic differentiation; Figure 1D).

### HBD4 Expression Increases in Response to Inflammatory Mediators and Promotes Migration of DPSC

**FIGURE 2:**
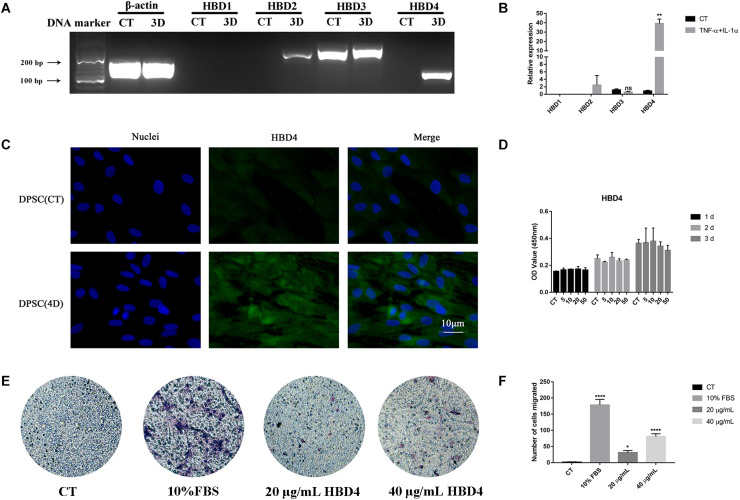
Expression of human beta defensin (HBD) in dental pulp stem cells (DPSC) and the biological activity of HBD4. **(A)** Expression of HBD1-4 in DPSC determined by qPCR and agarose gel electrophoresis. **(B)** Relative expression of HBD1-4 in CT and TNF-α + IL-1α stimulation group. **(C)** The expression of HBD4 in DPSC detected by immunofluorescence (fluorescence microscope, 1000 ×). Blue fluorescence indicates the nucleus stained by DAPI, and green fluorescence indicates HBD4 expression. **(D)** Effect of HBD4 on cell viability in DSPC. DPSC were treated with different concentrations of HBD4 (0, 5, 10, 20, and 50 μg/mL) for 1, 2, and 3 days before measuring cell viability using the CCK-8 assay. **(E)** Representative images of migrated cells in control, 20 μg/mL HBD4, 40 μg/mL HBD4, and 10% FBS groups in transwell assay. **(F)** Relative comparison of migrated cell numbers among the four groups described in **(E)**. All experiments were performed three times. ns, no significance; ^∗^*p* < 0.05; ^∗∗^*p* < 0.01; ^****^P < 0.0001 (CT, control; 3D, 3 days; 4D, 4 days).

mRNA expression of HBD4 was significantly upregulated 3 days after stimulation with tumor necrosis factor-alpha (TNF-α; 10 ng/mL) and interleukin (IL)-1α (10 ng/mL) compared to HBD1-3 (Figures 2A,B). Similarly, HBD4 expression increased in the cytoplasm of DPSC 4 days after combined stimulation with TNF-α and IL-1α (Figure 2C). While HBD4 did not affect the viability of DPSC (Figure 2D), it promoted the migration of DPSC in a concentration-dependent manner (Figures 2E,F).

### HBD4 Inhibits LPS-Mediated Inflammation in DPSC

**FIGURE 3:**
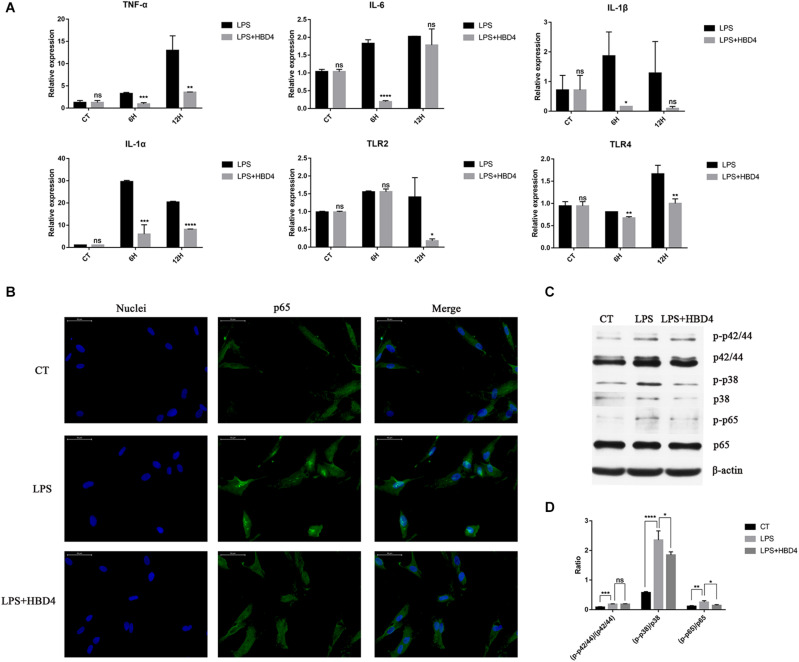
Anti-inflammatory effects of HBD4 in dental pulp stem cells (DPSC). **(A)** Effects of HBD4 on lipopolysaccharide (LPS)-induced expression of inflammatory mediators in DPSC. Statistical comparisons are between LPS alone vs. LPS with HBD4 at the same time point in each group. **(B)** The effect of HBD4 on the distribution of p65 in DPSC detected by immunofluorescence (fluorescence microscope, 1000 ×). Blue fluorescence indicates the nucleus stained by DAPI, and green fluorescence marks p65 expression. **(C)** Sodium dodecyl sulfate-polyacrylamide gel electrophoresis of cellular proteins. **(D)** Quantification of the band density observed in **(C)** determined using Image J software. All experiments were performed three times. *P < 0.05; **P < 0.01; ***P < 0.001; ****P < 0.0001; ns, no significance (CT, control; 6H, 6 h; 12H, 12 h).

HBD4 inhibited the LPS-mediated upregulation of TNF-α, IL-6, IL-1α, IL-1β, Toll-like receptor (TLR) 2, and TLR4 (Figure 3A). To explore its underlying mechanism, we examined whether HBD4 inhibited LPS-induced inflammation-related signaling pathways, including the NF-κB signaling pathway. We found p65 was mainly distributed in the cytoplasm of DPSC but entered the nucleus following LPS stimulation for 3 h (Figure 3B). However, in DPSC pretreated with HBD4, p65 was retained in the cytoplasm following LPS stimulation (Figure 3B). This suggests the NF-κB signaling pathway was inhibited by HBD4. Levels of phosphorylated p38 MAPK and p65 NF-κB were also reduced if DPSC were pre-incubated with HBD4 and challenged with LPS (Figures 3C,D).

### HBD4 Promotes Mineralizing Cell Phenotype Differentiation of DPSC

**FIGURE 4:**
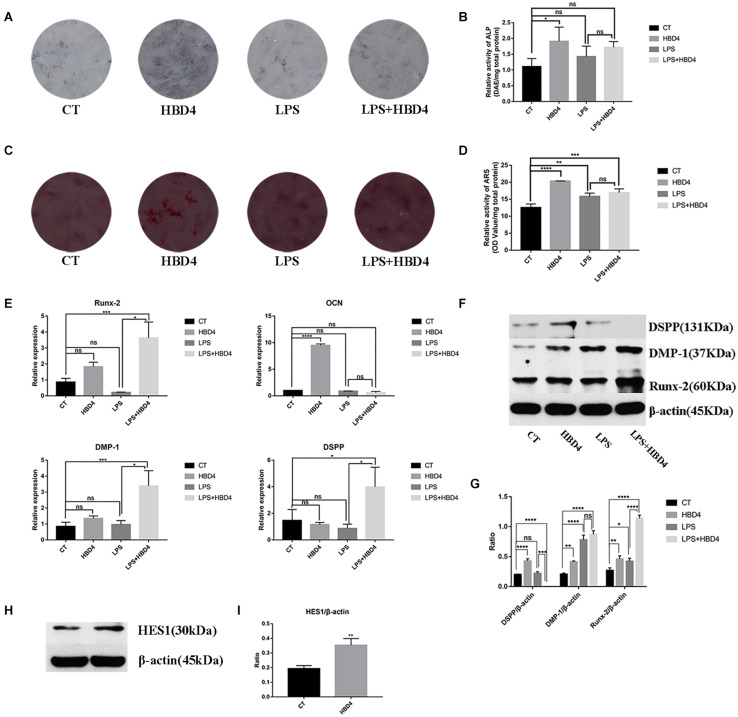
Effects of HBD4 on mineralizing cell phenotype differentiation in dental pulp stem cells (DPSC). DPSC were treated with HBD4 (10 μg/mL) and lipopolysaccharide (LPS, 1 μg/mL). Alkaline phosphatase (ALP) staining at 7 days **(A,B)** and alizarin red staining (ARS) staining at 21 days **(C,D)** of DPSC after HBD4 and LPS treatment. Effects of HBD4 on osteoblast/odontoblast gene **(E)** and osteoblast/odontoblast protein **(F,G)** expressions of DPSC in control and inflammatory microenvironments at 21 days. Effects of HBD4 on **(H,I)** HES1 expression detected by western blot. All experiments were performed three times. *P < 0.05; **P < 0.01; ***P < 0.001; ****P < 0.0001; ns, no significance (CT, control).

The effects of HBD4 on early mineralizing cell phenotype differentiation of DPSC were assessed by ALP staining on day 7. The ALP activity of DPSC treated with HBD4 was higher than that of controls (Figures 4A,B). The effects of HBD4 on late osteoblast differentiation of DPSC were assessed by ARS. The stain color was deeper in the HBD4 group, indicating that the degree of mineralization was increased (Figures 4C,D).

We also evaluated the expression of osteoblast/odontoblast markers in DPSC on day 21. In terms of osteoblast markers, HBD4 increased the mRNA expression of osteocalcin (OCN) in the absence of LPS and that of the Runt-Related Transcription Factor 2 (Runx-2) in the presence of LPS. Regarding odontoblast markers, DSPP and DMP-1 mRNA levels increased in the LPS + HBD4 group vs. the LPS group (Figure 4E). HBD4 also upregulated the protein expression level of odontoblast markers (DSPP, DMP-1) in the absence of LPS and that of Runx-2 with/without LPS (Figures 4F,G). Finally, we found increased protein expression of HES1 in the HBD4 group, indicating the Notch signaling pathway may mediate the effect of HBD4 on mineralizing cell phenotype differentiation (Figures 4H,I).

### *In vivo* Response to Dental Pulp Capping With HBD4 and MTA

**FIGURE 5:**
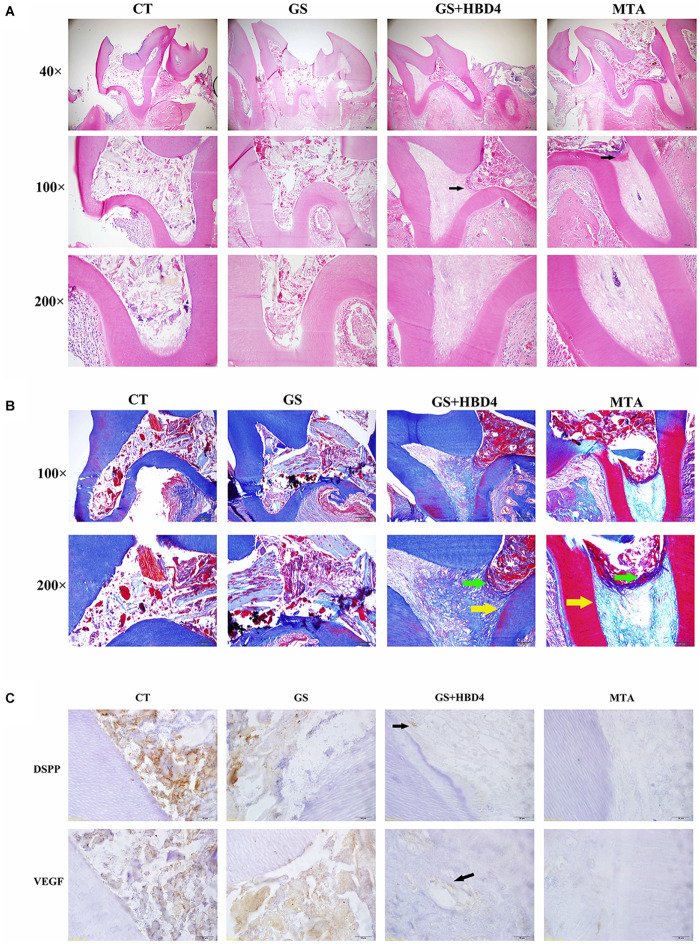
Effects of HBD4 in a rat model of reversible pulpitis. **(A)** HE staining, **(B)** Masson staining, and **(C)** DSPP/VEGF immunohistochemical staining of the maxillary first molar of Wistar rats at week 8 after the application of different pulp capping materials (optical microscope, 1000x). CT, control group; GS, gelatin sponge group; GS + HBD4, gelatin sponge and HBD4 group; MTA, mineral trioxide aggregate group.

The results of our pulp capping surgeries performed in Wistar rats are summarized in Table 2. Based on HE staining of the maxillary first molar at week 8 after the surgery, we found necrotic pulp tissue in the control and GS groups filled the whole pulp cavity and root canal (Figure 5A). Meanwhile, in rats treated with MTA or GS with HBD4, the necrotic and inflammatory tissues were limited and a newly-formed dentin-like barrier structure could be seen (note: above the barrier was the necrotic layer and below was the fibrous pulp tissue; Figure 5A). Similarly, Masson staining of the maxillary first molar at week 8 after surgery showed necrotic pulp tissue filled the root canal in the control and GS groups, while a newly-formed dentin-like barrier structure (Figure 5B, green arrow) and collagen fibrous pulp tissue (light blue staining) were observed in MTA-treated animals and those treated with both GS and HBD4. Furthermore, a mature hard tissue barrier (red staining) could be seen on the inner side of the root canal wall around the dentin-like barrier in the GS + HBD4 and MTA groups (Figure 5B, yellow arrow). No significant difference in the hard tissue formation and inflammatory cell responses was observed among the two groups (*P* > 0.05) (Table 3). Finally, immunohistochemical staining of DSPP and VEGF in the first molar of Wistar rats at week 8 after the surgery showed non-specific staining in the control and GS groups (Figure 5C). Meanwhile, some odontoblast-like cells were observed near the root canal wall in rats treated with both GS and HBD4, with positive DSPP staining, and some VEGF-positive cells around the lumen in the dental pulp tissue, indicative of neovascularization (Figure 5C). In contrast, only light immunoreactivity was detected in the pulp tissue of the MTA group (Figure 5C).

**TABLE 2:** The recorded results for pulp capping experiment.

	Survival rate	Filling retention	Dentin bridge formation	Pulp inflammation control	
				Yes	No
CT (*n* = 15)	15/15	12/15	0/15	0/15	15/15
GS (*n* = 5)	5/5	3/5	0/5	0/5	5/5
GS + HBD4 (*n* = 5)	5/5	4/5	3/5	3/5	2/5
MTA (*n* = 5)	5/5	4/5	4/5	4/5	1/5

**TABLE 3:** Occurrences of the evaluated histologic parameters in each group.

Scores	Groups		
	GS + HBD4(*n* = 5)	MTA(*n* = 5)	*P*-value
**Hard tissue formation**			
1 = Heavy: hard tissue deposition as complete and continuous dentin bridge	3	4	0.69
2 = Moderate: hard tissue formation as incomplete and discontinuous dentin bridge	0	0	
3 = Slight: a layer of scattered and foggy hard tissue deposition	0	0	
4 = No hard tissue deposition	2	1	
**Inflammatory cell response**			
1 = Absent or few inflammatory cells	0	0	0.69
2 = Mild: inflammatory cells only next to dentin bridge or area of pulp exposition	3	4	
3 = Moderate: inflammatory cells are observed in the part of root pulp	0	0	
4 = Severe: all root pulp	2	1	

## Discussion

There are some disadvantages in the clinical application of calcium silicate bioceramic materials, such as MTA: MTA can discolor teeth and is not suitable for pulp capping treatment of anterior teeth (Bogen et al., 2008). So far, there are many reports about the antibacterial activity of MTA, but the conclusions are different. The reason may be related to the difference of experimental design, detection methods and strains (Hernandez-Delgadillo et al., 2017; Shin et al., 2017; Huang et al., 2019). Moreover, their restorative effect on dental pulp do not follow the natural process of pulp-dentin complex. Therefore, anti-inflammatory and anti-bacterial compounds, such as HDB4, could have useful applications for those with pulpitis (Pazgier et al., 2006; Semple et al., 2010). We (and others) have previously shown HBD4 has good antibacterial activity against some of the common pathogenic bacteria associated with pulpitis, such as *Porphyromonas gingivalis* and *Enterococcus faecalis* (Lee and Baek, 2012; Zhai et al., 2019). We also showed HBD4 promoted the osteogenic/odontogenic differentiation of SHED (Zhai et al., 2019). In this study, we further explored whether HBD4 could be used in the vital pulp preservation of permanent teeth.

In permanent teeth, bacteria and endotoxins may affect odontoblasts as the first line of defense and later fibroblasts, lymphocytes, and DPSC (Cooper et al., 2010). DPSC play an important role in pulpal defense and are recruited from their niche, migrate to the affected site and then differentiate into odontoblasts to form reparative dentine (Shi and Gronthos, 2003). Considering that DPSC are the reserve cells for pulp injury and repair, we used LPS to stimulate DPSC to establish an inflammatory dental pulp cells model (Widbiller et al., 2018; Xue et al., 2018), and explored the anti-inflammatory and differentiation inducing function of HBD4 *in vitro*.

HBD4 was significantly upregulated in DPSC following stimulation with inflammatory factors, as previously observed in SHED (Zhai et al., 2019), and was mainly expressed in the cytoplasm of DPSC. HBD4 possessed strong anti-inflammatory activity *in vitro*. In addition, we found HBD4 promoted the differentiation of DPSC into odontoblasts/osteoblasts, similar to the function of HBD4 in SHED. In our previous study, it has been identified the osteoblast/odontoblast differentiation of SHED induced by HBD4 was related to the activation of Notch signaling pathway. Considering the controversial role of Notch signaling pathway in stem cell differentiation (Zhang et al., 2008; Zhou et al., 2018), we explored the role of Notch signaling pathway during DPSC differentiation induced by HBD4. Similar to SHED, the protein expression level of HES1 (key protein of Notch signaling pathway) in HBD4 group was significantly higher than that in the control group, which indicated the mineralizing cell phenotype differentiation of DPSC by HBD4 was related to Notch signaling pathway. However, the anti-inflammatory mechanism of HBD4 in DPSC differed from that observed in SHED: in SHED, HBD4 mainly exerts its anti-inflammatory action via p42/44 MAPK (Zhai et al., 2019), whereas we found HBD4 appears to act on p38 MAPK and the NF-κB signaling pathway in DPSC. The specific mechanism of inhibition of NF-κB pathway by HBD4 is not clear, but the inhibition effect by other members of HBD family can provide us with clues: C-terminal peptides within HBD3 could penetrate the outer membrane of LPS-treated RAW 264.7 macrophages and downregulate NF-κB-dependent inflammation by directly suppressing the degradation of phosphorylated-IκBα and by downregulating active NF-κB p65 (Lee et al., 2015). In the future research, we will study the specific mechanism of HBD4 inhibiting MAPK and NF-κB pathway. Furthermore, the LPS-stimulated DPSC model is not comprehensive enough to evaluate the dental pulp inflammation effect. We will also test the effect of HBD4 on other cell line in the dental pulp, such as fibroblasts and lymphocytes.

Using a rat model of reversible pulpitis, we found combining GS with HBD4 as the pulp capping material resulted in strong inflammation control. In these rats, odontoblasts were observed at the junction of the pulp tissue and root canal wall, and VEGF-positive cells were found around the lumen in the pulp tissue, indicating HBD4 has a role in pulp preservation. Despite these beneficial effects, the rate of inflammation control and dentin bridge formation in rats treated with the GS and HBD4 was lower than that observed in MTA-treated rats (no statistical difference). However, it is important to note that the application of HBD4 *in vivo* has not yet been optimized. In particular, the most appropriate scaffold material for the *in vivo* application of HBD4 should be determined. In our experiment, we used GS as the scaffold material; however, other biomaterials may be more suitable for use with HBD4. The optimal concentration of HBD4 for *in vivo* applications should also be determined in future. Although the animal model we used referred to previous literature reports (Zhu et al., 2019), the limitation was that the exposure time of LPS was too short to distinguish between traumatic pulpitis model and caries pulpitis model. In future experiments, we will increase the exposure time of LPS so as to further clarify the *in vivo* effect of HBD4 on pulpitis model for carious exposure.

In summary, HBD4 shows good anti-inflammatory activity *in vitro* and promotes mineralizing cell phenotype differentiation in DPSC. HBD4 also reduces the degree of inflammation *in vivo*, and preserves part of the living pulp tissue while inducing the formation of new odontoblasts. Together, our results suggest HBD4 has the potential for clinical application in the treatment of irreversible pulpitis. However, there are some outstanding questions. First, the half-life of HBD4 *in vivo* is relatively short, and therefore, we may need to improve its stability (e.g., by modifying its structure or using more appropriate scaffold materials) to facilitate its application in humans. Second, the precise target(s) of HBD4, as well as the upstream and downstream signaling pathways, that is responsible for its anti-inflammatory and pro-differentiation effects should be identified, to facilitate translation to the clinic.

## Data Availability Statement

The raw data supporting the conclusions of this article will be made available by the authors, without undue reservation, to any qualified researcher.

## Ethics Statement

The animal study was reviewed and approved by the Ethics Committee of the Peking University Health Science Center.

## Author Contributions

YZ conceptualized, planned, and performed all the experiments. YZ and YMZ wrote the manuscript. XY performed the experiments of quantitative polymerase chain reaction and western blot. LG contributed toward editing and proofreading the manuscript. YW was responsible for the overall project design and manuscript organization, revision, and finalization. All authors contributed to the article and approved the submitted version.

## Conflict of Interest

The authors declare that the research was conducted in the absence of any commercial or financial relationships that could be construed as a potential conflict of interest.
